# Antimicrobial susceptibility trends of *S.* Typhi and *S.* Paratyphi in a post-COVID-19 pandemic India, from a multicenter surveillance network

**DOI:** 10.1038/s41598-025-93170-7

**Published:** 2025-04-21

**Authors:** Nikhil Sahai, Jobin John Jacob, Dilesh Kumar Arunachalam, Bimal Kumar Das, Arti Kapil, Sangeeta Pandey, Shanta Dutta, Debjit Chakraborty, Arunima Sen Gupta, Madhu Gupta, Adarsh Bansal, Bhavini Shah, Veena Iyer, Chinjal Panchal, Shariqa Qureshi, Karnika Saigal, Diganta Saikia, Deepika Gupta, Savitha Nagaraj, Sanju Jose, Maria Thomas, Sangeetha Mohan, Balaji Veeraraghavan, Gagandeep Kang, Jacob John

**Affiliations:** 1 Department of Community Health, Christian Medical College Vellore, Vellore, India; 2 Department of Microbiology, Christian Medical College Vellore, Vellore, India; 3 Department of Microbiology, All India Institute of Medical Sciences, New Delhi, India; 4 ICMR-National Institute for Research in Bacterial Infections, Kolkata, India; 5 Epidemiology Division, ICMR-National Institute for Research in Bacterial Infections, Kolkata, India; 6 Bacteriology Division, ICMR-National Institute for Research in Bacterial Infections, Kolkata, India; 7 School of Public Health, Post Graduate Institute of Medical Education and Research, Chandigarh, India; 8 Neuberg Supratech Reference Laboratories, Ahmedabad, India; 9 Department of Public Health Programme, Indian Institute of Public Health Gandhinagar, Gandhinagar, India; 10 Division of Clinical Microbiology and Infectious Diseases, Chacha Nehru Bal Chikitsalaya, New Delhi, India; 11 Department of Pediatric Medicine, Chacha Nehru Bal Chikitsalaya, New Delhi, India; 12 Department of Microbiology, St. Johns Medical College Hospital, Bengaluru, India; 13 Department of Microbiology, Christian Medical College & Hospital, Ludhiana, India; 14 Wellcome Trust Research Laboratory, Christian Medical College Vellore, Vellore, India

**Keywords:** Enteric fever, Typhoid fever, Antimicrobial resistance, Typhi, Paratyphi, Paratyphoid fever, Bacterial infection, Antimicrobial resistance, Infectious-disease epidemiology

## Abstract

We conducted a multicenter surveillance study to identify changes in antimicrobial susceptibility patterns of *Salmonella* Typhi and *S.* Paratyphi in India since the COVID-19 pandemic began. We collected *S.* Typhi and *S.* Paratyphi isolates from blood or bone marrow culture-confirmed enteric fever cases at eight sites in seven cities across India between 2021 and 2024. We tested the antibiotic susceptibility of 1150 *S.* Typhi isolates and 265 *S.* Paratyphi isolates via disc diffusion and determined their minimum inhibitory concentrations (MICs) of ceftriaxone and azithromycin via broth dilution. We identified 18 *S.* Typhi isolates from Ahmedabad that were resistant to ceftriaxone, indicating a larger emergence of third-generation cephalosporin-resistant *S.* Typhi in Western India with a novel plasmid profile. Furthermore, we observed yearly increases in the mean, median and 90th percentile of azithromycin MICs for *S.* Typhi and *S.* Paratyphi isolates throughout India between 2021 and 2023. Finally, we found that only 0.70% of *S.* Typhi isolates and 1.13% of *S.* Paratyphi isolates exhibited susceptibility to ciprofloxacin. Our results indicate the necessity for a shift from ciprofloxacin in the treatment of enteric fever, and the importance of implementing long-term monitoring of resistance to alternative antibiotics such as azithromycin and ceftriaxone.

## Introduction

Enteric fever remains a major contributor to the worldwide burden of infectious diseases with 9.32 million cases estimated to have occurred in 2021 and approximately 107,000 deaths attributed to it^1^. These cases occur disproportionally in low- and middle-income countries in Asia and Africa, where the disease is endemic. In particular, South Asia has a high incidence (over 100 cases per 100,000 person-years), as shown by recent estimates from the surveillance for enteric fever in Asia Project^2^ and surveillance for enteric fever in India (SEFI)^3^ networks that encompass Nepal, Bangladesh, Pakistan, and India.

This burden is particularly alarming, considering the outbreaks of extensively drug-resistant (XDR) typhoid fever in Pakistan since 2016^4,5^. There is a threat of these strains spreading to neighboring countries, such as India, where drug resistance is already a risk owing to widespread misuse and self-medication with antibiotics^6^. Further changes in antibiotic resistance patterns among typhoid and paratyphoid cases may occur because of the COVID-19 pandemic, which saw a spike in the use of antibiotics such as azithromycin in comparison to that in 2019^7^. Some evidence of pandemic-related shifts may also be found in Pakistan, where *S.* Typhi isolates from patients who had typhoid in 2019 and again between 2022 and 2023 were found to have a higher ratio of XDR to multidrug-resistant (MDR) strains in the latter period^8^.

In such a situation, it is imperative to monitor the antimicrobial resistance patterns of *S.* Typhi and *S.* Paratyphi in India to ensure timely and effective control of enteric fever. Consequently, we conducted a second phase of the SEFI network surveillance at eight sites across seven cities between 2021 and 2024. In this study, we present the latest trends observed in this cross-country network regarding the antimicrobial susceptibility patterns of the collected *S.* Typhi and *S.* Paratyphi isolates.

## Results

### Culture results and demographic characteristics

A total of 270,228 blood and bone marrow cultures were conducted across the sites, of which 0.5% (n = 1229) were positive for *S.* Typhi and 0.1% (n = 272) were positive for *S.* Paratyphi A. While Tier 2 sites only enrolled hospitalized patients, 28.4% (n = 393) of enteric fever-positive patients (n = 1382) at Tier 3 sites were hospitalized. Most patients with enteric fever were under 15 years of age (62.9%, n = 944), and 40.9% (n = 614) were female. Tables 1 and 2 provide further site-wise details of the cultures and the patients’ demographics.

**Table 1:** Summary of blood and bone marrow cultures processed at surveillance for enteric fever in India (SEFI) sites.

Site*	Patients screened	Enrolled	Blood cultures conducted	Total enteric fever	*S*. Typhi	*S*. Paratyphi A
	N	n1 (% of N)	n2 (% of n1)	n (% of n2)	n (% of n2)	n (% of n2)
Tier 2						
CHD	37,735	1817 (4.8)	1770 (97.4)	63 (3.6)	37 (2.1)	26 (1.5)
VLR	41,130	2402 (5.8)	2311 (96.2)	56 (2.4)	41 (1.8)	15 (0.6)
Total	78,865	4219 (5.3)	4081 (96.7)	119 (2.9)	78 (1.9)	41 (1.0)

Site*	Total cultures	*S*. Typhi	*S*. Paratyphi A	Total enteric fever	Outpatient cases	Inpatient cases
	N1	n (% of N1)	n (% of N1)	N2 (% of N1)	n (% of N2)	N3 (% of N2)
Tier 3						
AIIMS	58,451	114 (0.2)	13 (0.02)	127 (0.2)	119 (93.7)	8 (6.3)
AMD	23,586	453 (1.9)	122 (0.5)	575 (2.4)	552 (96.0)	23 (4.0)
CMCL	9459	134 (1.4)	32 (0.3)	166 (1.8)	51 (30.7)	115 (69.3)
CNBC	29,655	320 (1.1)	36 (0.1)	356 (1.2)	224 (62.9)	132 (37.1)
KLK	38,213	44 (0.1)	16 (0.04)	60 (0.2)	26 (43.3)	34 (56.7)
STJ	31,999	86 (0.3)	12 (0.04)	98 (0.3)	17 (17.3)	81 (82.7)
Total	191,363	1151 (0.6)	231 (0.1)	1382 (0.7)	989 (71.6)	393 (28.4)

*CHD Chandigarh, *VLR* Vellore, *AIIMS* All India Institute of Medical Sciences New Delhi, *AMD* Ahmedabad, *CMCL* Christian Medical College Ludhiana, *CNBC* Chacha Nehru Bal Chikitsalya New Delhi, *KLK* Kolkata, *STJ* St. Johns Medical College Hospital, Bengaluru.

**Table 2:** Age and sex distributions of patients positive for *S.* Typhi and *S.* Paratyphi A.

Site*	Total N	Female n (% of N)	< 15 years n (% of N)
*S*. Typhi			
CHD	37	14 (37.8)	15 (40.5)
VLR	41	22 (53.7)	25 (61.0)
AIIMS	114	40 (35.1)	43 (37.7)
AMD	453	185 (40.8)	285 (62.9)
CMCL	134	53 (39.6)	60 (44.8)
CNBC	320	142 (44.4)	320 (100)
KLK	44	19 (43.2)	10 (22.7)
STJ	86	32 (37.2)	34 (39.5)
Total	1229	507 (41.3)	792 (64.4)
*S*. Paratyphi A			
CHD	26	19 (73.1)	15 (57.7)
VLR	15	6 (40.0)	7 (46.7)
AIIMS	13	4 (30.8)	4 (30.8)
AMD	122	44 (36.1)	71 (58.2)
CMCL	32	15 (46.9)	9 (28.1)
CNBC	36	14 (38.9)	36 (100)
KLK	16	2 (12.5)	4 (25.0)
STJ	12	3 (25.0)	6 (50.0)
Total	272	107 (39.3)	152 (55.9)

*CHD Chandigarh, *VLR* Vellore, *AIIMS* All India Institute of Medical Sciences New Delhi, *AMD* Ahmedabad, *CMCL* Christian Medical College Ludhiana, *CNBC* Chacha Nehru Bal Chikitsalya New Delhi, *KLK* Kolkata, *STJ* St. Johns Medical College Hospital, Bengaluru.

Three isolates were determined to be *S.* Paratyphi B variant Java via whole-genome sequencing analysis and hence were considered non-typhoidal^9^.

### Antimicrobial susceptibility profiles

Almost all *S.* Typhi isolates (99.3%) were non-susceptible to ciprofloxacin (Table 3), and *S.* Paratyphi A isolates had similar resistance patterns, with 98.9% of the isolates being non-susceptible. Furthermore, only 1.8% (n = 21) of *S.* Typhi isolates were MDR, that is, resistant to ampicillin, chloramphenicol, and sulfamethoxazole + trimethoprim. Of these, 10 were from Delhi and 11 were from Ahmedabad; no *S.* Paratyphi A isolates were found to be MDR.

**Table 3:** Proportion of *S.* Typhi and *S.* Paratyphi isolates susceptible to the antibiotic panel.

Region	Isolates (N)	Antibiotics* (% of N)					
		AMP	CHL	SXT	CFT	CIP	AZM
*S*. Typhi							
North	543	98.2	98.0	95.8	100	0.0	100
South	127	100	100	100	100	0.0	100
East	44	100	100	100	100	0.0	100
West	436	93.3	96.3	92.4	95.9	1.8	100
Total	1150	96.6	97.7	95.1	98.4	0.7	100
*S.* Paratyphi A							
North	102	99.0	99.0	100	100	0.0	99.0
South	27	100	100	100	100	0.0	100
East	16	100	100	100	100	0.0	100
West	120	100	100	100	100	2.5	100
Total	265	99.6	99.6	100	100	1.1	99.6

*AMP ampicillin, *CHL* chloramphenicol, *SXT* sulfamethoxazole + Trimethoprim, *CFT* ceftriaxone, *CIP* ciprofloxacin, *AZM* azithromycin.

We found that 18 *S.* Typhi isolates collected from Ahmedabad between Jun 1, 2022, and Apr 30, 2023, were resistant to the 3rd generation cephalosporin tested (ceftriaxone), along with ampicillin, sulfamethoxazole + trimethoprim, and ciprofloxacin, and were susceptible to azithromycin and chloramphenicol. Additionally, one *S.* Paratyphi A isolate collected from AIIMS Delhi in May 2023 was resistant to azithromycin (MIC = 64 µg/mL) and ciprofloxacin, while being susceptible to ampicillin, chloramphenicol, sulfamethoxazole + trimethoprim, and ceftriaxone.

### Genomic analysis

The whole-genome analysis of ceftriaxone-resistant *S.* Typhi isolates identified them as belonging to the H58 lineage (genotype 4.3.1), with further classification into the subgenotype 4.3.1.2.2. Comprehensive antimicrobial resistance gene profiling revealed the presence of *bla*_CTX-M-15_, *qnrS1, sul2, dfrA14,* and *tet(A),* all localized on an IncFIB(K) plasmid. Mutation analysis within the quinolone resistance-determining region (QRDR) identified a S83F substitution in *gyrA*, which is associated with reduced susceptibility to fluoroquinolones. A notable observation was the plasmid composition, which included three plasmids in *S*. Typhi isolates which have not been observed in previous studies. Among these, IncFIB(K) was the only plasmid carrying antimicrobial resistance genes, whereas IncFIB(pHCM2) and IncX1 did not encode any known resistance determinants.

### MIC analysis

**Fig. 1:**
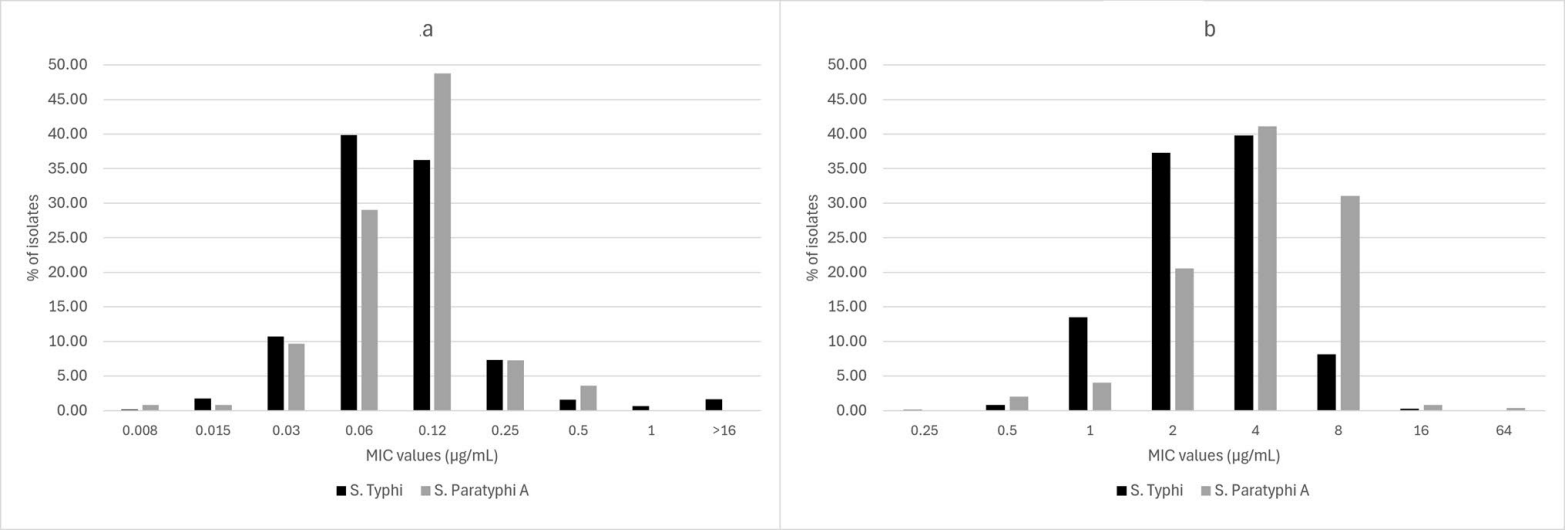
(**a**) Distribution of ceftriaxone minimum inhibitory concentration (MIC) values for *S.* Typhi (N = 1081) and *S.* Paratyphi A isolates (N = 248); (**b**) Distribution of azithromycin minimum inhibitory concentration (MIC) values for *S.* Typhi (N = 1080) and *S.* Paratyphi A isolates (N = 248).

The MIC values for ceftriaxone ranged from 0.008 to > 16 µg/mL among *S.* Typhi isolates (Table 4). Mean MICs were not calculated since endpoint MICs for ceftriaxone could not be determined due to high resistance. The MIC 50 and MIC 90 values were low and had little variation between regions, with overall values of 0.06 µg/mL and 0.25 µg/mL, respectively. Among *S.* Paratyphi A isolates, the range was narrower and ranged from 0.008 to 0.5 µg/mL (Table 4) with an overall low and susceptible mean of 0.12 µg/mL. There was little variation in the mean MIC between regions. The MIC 50 values were also similar across the regions, with an overall value of 0.12 µg/mL, while MIC 90 values were low and ranged from 0.12 µg/mL among isolates from the Southern and Eastern sites, to 0.25 µg/mL in the Northern and the Western sites. A distribution of the MIC values showed that 76.1% of *S.* Typhi isolates had ceftriaxone MICs of 0.06–0.12 µg/mL (Fig. 1a). This range also accounted for 77.8% of all *S.* Paratyphi A isolates.

**Table 4:** Summary of the minimum inhibitory concentrations (MIC) of ceftriaxone and azithromycin for *S.* Typhi and *S.* Paratyphi isolates.

Organism	Region	Isolates (N)	MIC range (µg/mL)	Mean MIC (µg/mL)	MIC 50* (µg/mL)	MIC 90* (µg/mL)
Ceftriaxone						
*S.* Typhi	North	543	0.015–1	–	0.12	0.25
	South	127	0.008–0.5	–	0.06	0.12
	East	44	0.03–0.12	–	0.06	0.12
	West	367	0.015–> 16	–	0.06	0.25
	Total	1081	0.008–> 16	–	0.06	0.25
*S.* Paratyphi A	North	100	0.015–0.5	0.12	0.12	0.25
	South	27	0.008–0.5	0.10	0.12	0.12
	East	16	0.03–0.25	0.08	0.06	0.12
	West	105	0.03–0.5	0.12	0.12	0.25
	Total	248	0.008–0.5	0.12	0.12	0.25
Azithromycin						
*S.* Typhi	North	543	0.50–16	3.58	4	8
	South	127	0.5–8	3.29	4	4
	East	44	1–8	3.11	4	4
	West	366	0.25–8	2.54	2	4
	Total	1080	0.25–16	3.17	2	4
*S.* Paratyphi A	North	100	1–64	5.95	4	8
	South	27	0.5–16	5.72	4	8
	East	16	0.5–8	4.41	4	8
	West	105	0.5–8	3.95	4	8
	Total	248	0.5–64	4.98	4	8

*MIC 50 and MIC 90 represent the 50th percentile and 90th percentile, respectively, of the MIC values for *S.* Typhi and *S.* Paratyphi A isolates.

*Salmonella* Typhi isolates from Western India had a slightly lower mean azithromycin MIC of 2.54 µg/mL as compared to the other regions whose means ranged from 3.11 µg/mL in the East to 3.58 µg/mL in the North (Table 4). The MIC 50 values had similar patterns with a lower MIC 50 among isolates from Western India (2 µg/mL), as compared to the other regions (4 µg/mL). Further, the MIC 90 value for Northern India was higher (8 µg/mL) as compared to other regions (4 µg/mL). Overall, the MICs ranged from 0.25 to 16 µg/mL. The mean MICs were higher among *S.* Paratyphi A isolates and ranged from 3.95 µg/mL in Western India to 5.95 µg/mL in the North. The MIC 50 values were similar to the values for *S.* Typhi isolates, except for Western India where the median was higher among *S.* Paratyphi A isolates (4 µg/mL). The overall MIC 90 value (8 µg/mL) was double the overall value of *S.* Typhi isolates (4 µg/mL). One *S.* Paratyphi A isolate from North India (AIIMS Delhi) had a MIC of 64 µg/mL and was interpreted as resistant by adapting Clinical and Laboratory Standards Institute’s (CLSI) breakpoints for *S.* Typhi^10^, while the other *S.* Paratyphi A isolates had MICs which ranged from 0.5 to 16 µg/mL. The MIC distributions of the isolates are shown in Fig. 1b.

A time series of azithromycin MICs of the *S.* Typhi isolates collected showed that the mean MICs increased each year from 2021 to 2023 across all four regions; the overall mean increased from 2.28 µg/mL in 2021 to 4.01 µg/mL in 2023. The overall MIC 50 values for *S.* Typhi isolates also increased from 2 µg/mL in 2021 to 4 µg/mL in 2023, while MIC 90 values increased from 4 µg/mL in 2021 to 8 µg/mL in 2023 (Table 5). We found similar results among *S.* Paratyphi A isolates from North India where the mean MIC increased from 3.50 µg/mL in 2021 to 8.11 µg/mL in 2023. Further, *S.* Paratyphi A isolates from Western India showed a mean MIC of 3.00 µg/mL in 2021 which was 4.57 µg/mL by 2023. The overall MIC 50 value also rose from 4 µg/mL in 2021 to 8 µg/mL in 2023 (Table 5). There were insufficient *S.* Paratyphi isolates in the South and East for all 3 years to determine their MIC trends.

**Table 5:** Summary of the 50th (MIC 50) and 90th (MIC 90) percentiles of azithromycin minimum inhibitory concentrations (MIC) for *S.* Typhi and *S.* Paratyphi A isolates, by year and region.

Region	Year												Overall			
	2021				2022				2023							
	Isolates (n)	% of n sus.	MIC 50	MIC 90	Isolates (n)	% of n sus.	MIC 50	MIC 90	Isolates (n)	% of n sus.	MIC 50	MIC 90	Isolates (n)	% of n sus.	MIC 50	MIC 90
*S. Typhi*																
North	52	100	2	4	261	100	4	4	223	100	4	8	536	100	4	8
South	19	100	2	4	68	100	2	4	40	100	4	6	127	100	4	4
East	0	–	–	–	27	100	2	4	17	100	4	4	44	100	4	4
West	72	100	2	4	223	100	2	4	71	100	4	4	366	100	2	4
Total	143	100	2	4	579	100	2	4	351	100	4	8	1073	100	2	4
*S. Paratyphi*																
North	12	100	4	4	52	100	4	8	36	97.2	8	8	100	99.0	4	8
South	10	100	4	8	14	100	4	8	3	100	4	16	27	100	4	8
East	0	–	–	–	13	100	4	8	3	100	4	4	16	100	4	8
West	23	100	2	4	61	100	4	8	21	100	4	8	105	100	4	8
Total	45	100	4	8	140	100	4	8	63	98.4	8	8	248	99.6	4	8

The proportions of isolates susceptible (sus) to azithromycin as per Clinical and Laboratory Standards Institute guidelines are also included. All MIC measures are in µg/mL.

## Discussion

The second phase of surveillance undertaken by the SEFI network provided an overview of antimicrobial susceptibility and epidemiological changes among *S.* Typhi and *S.* Paratyphi A isolates in India since the COVID-19 pandemic began. Our network detected a novel genotype of ceftriaxone-resistant *S.* Typhi in Western India. Furthermore, we found a yearly increasing trend in the azithromycin MICs among the isolates tested across India between 2021 and 2023. We also discovered that nearly all isolates tested (99.3% of *S.* Typhi isolates and 98.9% of *S.* Paratyphi A isolates) were non-susceptible to ciprofloxacin.

The proportion of susceptibility among the isolates to ciprofloxacin was similar to that found in SEFI’s first phase, where > 97% of *S.* Typhi and *S.* Paratyphi A isolates were non-susceptible^11^. Our results reiterate the need to phase out ciprofloxacin from enteric fever treatment regimens. Furthermore, > 95% of *S.* Typhi and > 99% of *S.* Paratyphi A isolates were susceptible to the previous generation of first-line antibiotics, that is, ampicillin, chloramphenicol, and sulfamethoxazole + trimethoprim, and only 1.8% were non-susceptible to all three (MDR), which was similar to the findings from the first phase^11^. These results show that the proportion of MDR cases in India has remained consistently low since SEFI’s first phase began in 2017, which may again be due to recent antibiotic prescription patterns in India that favor the use of cephalosporins and cephalosporin-fluoroquinolone combinations^12,13^.

This use of cephalosporins may contribute to the spread of 3rd generation cephalosporin-resistant *S.* Typhi isolates, especially in Western India. The 18 isolates found in Ahmedabad in Western India join previous sporadic reports of ceftriaxone resistance across North India^14^, South India^15^, East India^16^, and slightly south of Ahmedabad in Mumbai^17^. Genomic analysis of these isolates (genotype 4.3.1.2.2) revealed three plasmids that distinguished them from previous cephalosporin-resistant *S.* Typhi isolates found in India, i.e. IncFIB(K) (carrying antimicrobial resistance genes), IncFIB(pHCM2) and IncX1. These *S.* Typhi isolates may have acquired such plasmids from other Enterobacteriaceae, as seen previously^18^, and could be further selected for due to antibiotic usage pressure which increases the risk of a pan-Indian wave of 3rd generation cephalosporin-resistant *S.* Typhi in the coming years.

Our surveillance also revealed an increase in mean MICs, and MIC 50 and MIC 90 values of azithromycin for *S.* Typhi isolates across all regions between 2021 and 2023, and also among *S.* Paratyphi A isolates except at the Southern and Eastern sites, where there were insufficient isolates to observe trends. This was similar to the findings of a study conducted at AIIMS Delhi, which found increasing median azithromycin MICs among *S.* Typhi isolates from 2007 to 2016^19^ and might indicate an increased risk of azithromycin resistant strain outbreaks in the coming years. However, unlike in our study, they were unable to identify a trend among *S.* Paratyphi A isolates, possibly because of their low numbers. We also found that the mean MICs, and MIC 50 and MIC 90 values for azithromycin among *S.* Typhi isolates in 2021, 2.28 µg/mL, 2 µg/mL, and 4 µg/mL respectively, were lower than the same measures from SEFI’s first phase i.e., 4.98 µg/mL, 4 µg/mL, and 8 µg/mL respectively^11^. A similar comparison could also be made for the mean MIC, and MIC 90 values for azithromycin among *S.* Paratyphi isolates between the two phases of SEFI. This may have been due to the reduced selection pressure caused by a sharp drop in cases (over 1 million fewer cases) of typhoid fever since the COVID-19 pandemic began in 2020 compared with that in 2019^20,21^. However, even with fewer cases, the mean MICs, and MIC 50 and MIC 90 values continued to increase until the end of SEFI’s second phase of surveillance in early 2024, which could be indicative of undetected enteric fever cases treated with azithromycin. Further monitoring of antimicrobial susceptibility patterns in *S.* Typhi and *S.* Paratyphi A is required to better understand this trend and control the risk of azithromycin-resistant strain outbreaks.

A limitation of our study was the lack of data from rural India, which may have presented isolates with different antimicrobial susceptibility patterns. Furthermore, sentinel surveillance sites may not be wholly representative of their regions, and larger diagnostic laboratory networks, such as ours in Ahmedabad, may help to better understand trends in different parts of India, which is evident from the ceftriaxone-resistant *S.* Typhi isolates we detected in Ahmedabad and the large number of enteric fever cases we identified at the site. However, utilizing laboratory networks for routine surveillance may be challenging in some regions, as found by Sonal et al.^22^, who are part of the WHO-IAMM network for surveillance of antimicrobial resistance (WINSAR) in Delhi and face numerous obstacles related to infrastructure, manpower, and training to obtain quality data on pathogenic organisms and their antimicrobial susceptibility patterns from collaborating laboratories.

Surveillance networks in India, such as ours, focus on limited sets of singular pathogens and provide valuable data on their burden and antimicrobial resistance. The second phase of surveillance for enteric fever under SEFI demonstrated the need to phase out the use of fluoroquinolones in cases of enteric fever. Furthermore, it highlights the need for continuous and long-term monitoring of *S.* Typhi and *S.* Paratyphi A isolates in India to control rising ceftriaxone and azithromycin resistance. However, developing such extensive monitoring networks requires greater collaboration between governmental health agencies and private diagnostic centers, along with capacity-building initiatives, to ensure that all participating laboratories are well equipped for the task. Data from our study and similar future surveillance efforts will enable the effective deployment of control measures against enteric fever in India, such as Typhoid Conjugate Vaccines (TCVs) which are cost-saving in areas with high-burden^23^, and may even lead to an overall reduction of antimicrobial usage in the country due to a decline in typhoid cases^24^.

## Methods

### Ethics approval and consent to participate

Details of a patient’s course of hospitalization at a study site were collected after obtaining written informed consent from the patient or from the patient’s legal guardian, where applicable.

The study followed the applicable local and national guidelines and regulations for research involving human participants. It was approved by the Indian Government’s Health Ministry Screening Committee (TDR/692/2017-ECD-II, dated 7th July 2017) and institutional review boards of participating sites:

- Chandigarh—Postgraduate Institute of Medical Education and Research, Chandigarh, Institutional Ethics Committee (reference number PGI/IEC/2021/000612 dated May 3, 2021); Government Medical College and Hospital, Sector 32, Chandigarh Institutional Ethics Committee (reference number GMCH/IEC/2021/633R/368 dated September 24, 2021).
- Vellore—Christian Medical College, Vellore, Institutional Review Board (minute number 13489 (OBSERVE) dated October 28, 2020).
- Ludhiana—Christian Medical College and Hospital, Ludhiana, Institutional Ethics Committee (reference number IECCMCL/BMHR-06-362-21/Ren-Apprvl/Microbiology, dated June 24, 2021).
- Delhi—All India Institute of Medical Sciences, New Delhi, Institute Ethics Committee (reference number IEC-562/03.11.2017, RP-13/2017, dated November 6, 2017, extension on April 5, 2021); Chacha Nehru Bal Chikitsalaya, Delhi (reference number F.1/IEC/CNBC/17/04/2021/Protocol no. 99/13159 dated November 2, 2021).
- Ahmedabad—Indian Institute of Public Health, Gandhinagar, Institutional Ethics Committee (reference number TRC/2020-21/18, dated November 15, 2021).
- Kolkata—The Calcutta Medical Research Institute, Kolkata, Institutional Ethics Committee (reference number IEC/02/2022/APRV/16 dated October 27, 2022); Fortis Hospital, Kolkata, Ethics Committee (IEC protocol submission number IEC/2022/OAS/02 dated June 15, 2022); Ruby General Hospital Ltd., Kolkata, Institutional Ethics Committee (format number IEC-RGH/KOL/2021/002/CS dated March 28, 2022).
- Bengaluru—St. Johns Medical College & Hospital, Bengaluru, Institutional Ethics Committee (reference number IEC/1/1272/2021, dated November 18, 2021)

### Study setting

Data were collected from eight sites representing the Northern, Southern, Eastern, and Western regions of India. Of these, the Chandigarh and Vellore sites collected and reported *S.* Typhi and *S.* Paratyphi isolates as part of a hybrid surveillance setup (Tier 2) as previously described^25^. The remaining six were lab-based surveillance setups that involved tertiary care hospitals and regional reference laboratories, similar to the Tier 3 surveillance in SEFI’s first phase^26^. Study hospitals were tertiary care centers unless otherwise specified in the subsequent section. These centers primarily serve their local populations and offer some referral services to patients from nearby towns and villages. Additionally, the Christian Medical College, Vellore, and the All India Institute of Medical Sciences, New Delhi, have broader areas from which they attract referrals. Patients at the study hospitals are from a variety of age-groups and socio-economic strata.

Northern sites:

- Chandigarh (CHD): Civil Hospital Sector-45 (secondary care), Government Medical College and Hospital Sector 32 (tertiary care), and Government Multispecialty Hospital Sector 16 (tertiary care) were part of the surveillance network. It should be noted that Civil Hospital Sector-45 was a secondary care center and would primarily serve its nearby wards. The Post-Graduate Institute of Medical Education and Research (PGIMER) Chandigarh was the coordinating center for the site and processed all blood samples collected.
- All India Institute of Medical Sciences (AIIMS), New Delhi.
- Chacha Nehru Bal Chikitsalaya (CNBC), New Delhi; this is a pediatric specialty hospital that does not treat those who are 15 years of age or older.
- Christian Medical College, Ludhiana (CMCL).

Western site:

- Ahmedabad (AMD): CIMS Hospital, SAL Hospital and Medical Institute, Apollo Hospital International Ltd. Gandhinagar and Neuberg Supratech Reference Laboratories (NSRL) provided laboratory-based surveillance of the site. The three hospitals provided up-to-tertiary care; NSRL acted as a reference laboratory for healthcare facilities in the region and, in collaboration with the Indian Institute of Public Health, Gandhinagar, was the coordinating center for this site.

Eastern site:

- Kolkata (KLK): The Calcutta Medical Research Institute, Chittaranjan National Cancer Institute, Fortis Hospital, Anandpur, and Ruby General Hospital provided laboratory-based surveillance of the site. All four hospitals provided up-to-tertiary care. The surveillance was coordinated by the ICMR- National Institute for Research in Bacterial Infections (NIRBI, previously the National Institute of Cholera and Enteric Diseases), Kolkata.

Southern sites:

- Vellore: The Christian Medical College (CMC), Community Health and Development Unit (CHAD), and Low-Cost Effective Care Unit (LCECU) were part of the surveillance network. It should be noted that CHAD and LCECU provide primary and secondary care to their local populations. CMC Vellore processed all the blood samples collected.
- St. John’s Medical College Hospital (STJ), Bengaluru.

**Fig. 2:**
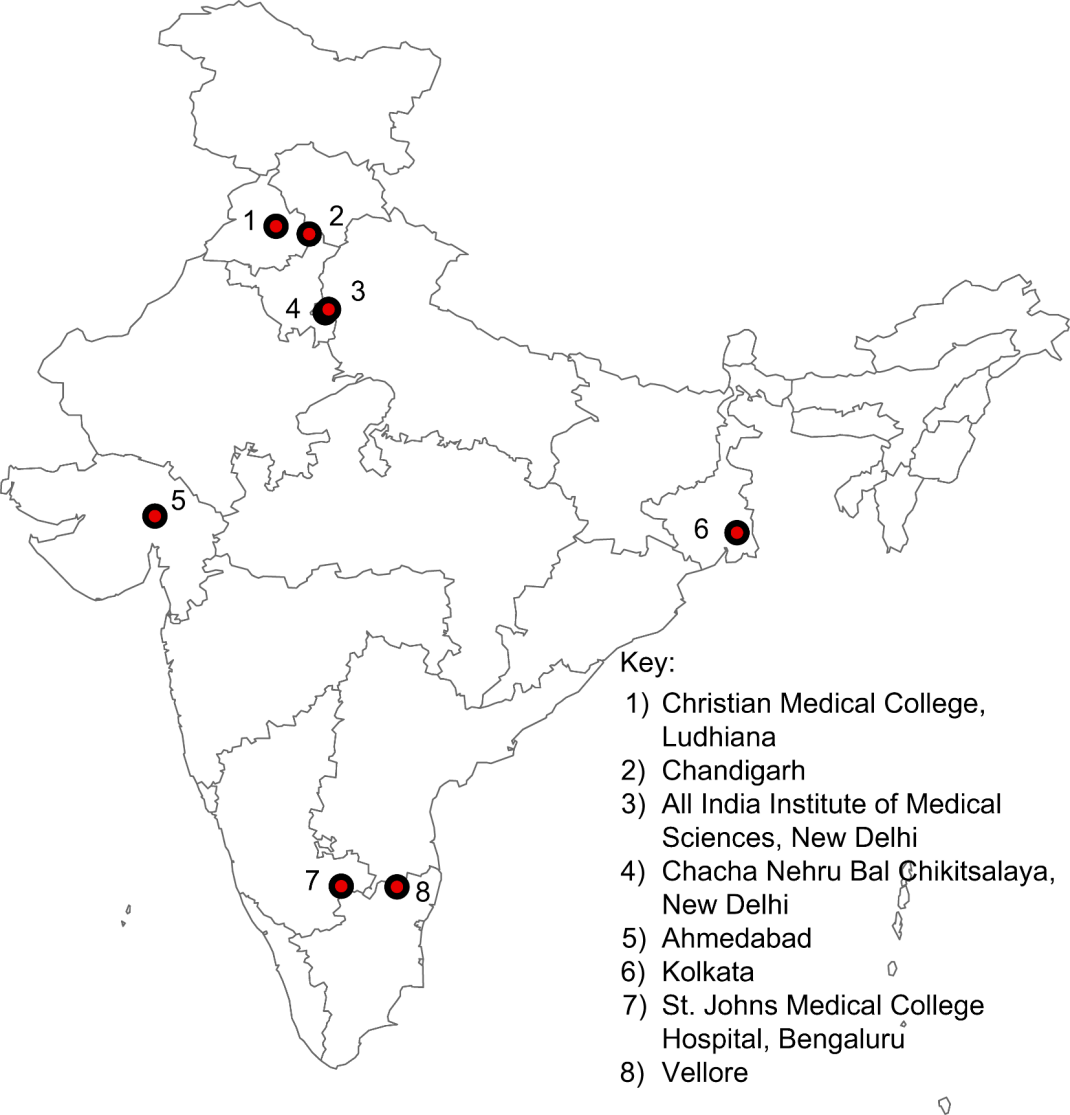
Geographical locations of sites participating in the second phase of the Surveillance for Enteric Fever in India study. The map was created using ArcGIS ver. 10.8 (https://www.arcgis.com/index.html).

Figure 2 shows the geographical locations of the sites.

### Inclusion and exclusion criteria

At Tier 2 sites, consenting patients who were ≥ 6 months of age, from the site’s catchment areas, and admitted with a febrile illness at a study hospital were recruited and had blood samples collected, which were subsequently cultured. Patients with known diagnoses of malignancy or those who were not Indian citizens were excluded. At Tier 3 sites, cultures of blood or bone marrow samples collected from patients were monitored via routinely maintained laboratory registers at each participating center. The decision to conduct either a blood or bone marrow culture was taken by a patient’s treating physician. There were no exclusion criteria for Tier 3. The surveillance periods for each site are listed in Table 6.

**Table 6:** Participating sites in the 2nd phase of the Surveillance for Enteric Fever in India study and their surveillance periods.

Site	Tier	Surveillance period
Chandigarh	Tier 2	Oct 4, 2021–Oct 3, 2023
Vellore	Tier 2	Mar 29, 2021–Feb 28, 2023
AIIMS New Delhi	Tier 3	May 25, 2021–May 25, 2023
Ahmedabad	Tier 3	May 15, 2021–May 15, 2023
CNBC New Delhi	Tier 3	Oct 22, 2021–Oct 21, 2023
St. Johns Medical College Hospital, Bengaluru	Tier 3	Jun 1, 2021–May 31, 2023
CMC Ludhiana	Tier 3	Feb 13, 2022–Feb 23, 2024
Kolkata	Tier 3	Jan 1, 2022–Aug 31, 2023

### Lab processes

Ahmedabad, Chandigarh, and Vellore used automated BACTEC systems for blood and bone marrow cultures, whereas St. Johns, Bengaluru, and Kolkata used automated BacT/Alert systems. CNBC Delhi and CMC Ludhiana had automated BACTEC systems, but conducted either automated or conventional blood cultures. AIIMS Delhi primarily conducts conventional cultures, with approximately 5% conducted using an automated BACTEC machine. Each site stored *S.* Typhi and *S.* Paratyphi isolates at − 20 °C or less. Of the isolates collected, 94.1% (n = 1415) were transferred to the CMC Vellore Microbiology Laboratory for re-identification through standard biochemical assays followed by serotyping using commercial antisera (BD Difco, USA) in accordance with the Kauffmann-White classification system, as described in the Manual of Clinical Microbiology^27^.

Kirby-Bauer disc diffusion (DD) assays were performed for ciprofloxacin (5 µg), pefloxacin (5 µg), ampicillin (10 µg), ceftriaxone (30 µg), co-trimoxazole (1.25/23.75 µg), azithromycin (15 µg) and chloramphenicol (30 µg). MICs were determined for azithromycin (0.12–128 µg/mL) and ceftriaxone (0.008–16 µg/mL) via broth microdilution which were utilized to reconfirm any resistance found via DD tests. Antimicrobial susceptibility test results were interpreted as per CLSI guidelines^10^. The 50th percentile (MIC 50) and 90th percentile (MIC 90) MIC values were also calculated for ceftriaxone and azithromycin. The reference isolate for DD tests involving ampicillin (10 µg; 15–22 mm), co-trimoxazole (1.25/23.75 µg; 23–29 mm), chloramphenicol (30 µg; 21–27 mm), ceftriaxone (30 µg; 29–35 mm), ciprofloxacin (5 µg; 29–37 mm), and pefloxacin (5 µg; 25–33 mm), was American Type Culture Collection (ATCC) *E. coli* 25922, while the reference for azithromycin (15 µg; 21–26 mm) DD tests was *S. aureus* 25923. Broth microdilution testing for azithromycin (0.50–2 µg/mL) used ATCC *S. aureus* 29213 as the reference, and ATCC *E. coli* 25922 for ceftriaxone (0.03–0.12 µg/mL).

The *S*. Paratyphi B isolates, and ceftriaxone resistant *S.* Typhi isolates were selected for whole-genome sequencing. Genomic DNA was extracted using the QIAamp® Mini Kit (QIAGEN, Hilden, Germany) following the manufacturer’s protocol. DNA purity was evaluated using a Nanodrop One spectrophotometer (Thermo Fisher, Waltham, USA), while precise quantification was performed using a Qubit Fluorometer with the dsDNA HS Assay Kit (Life Technologies, Carlsbad, USA). For short-read sequencing, genomic DNA was fragmented, and paired-end libraries were constructed using the Illumina Nextera DNA Flex Library Kit and Nextera DNA CD Indexes (Illumina, Massachusetts, USA). Equimolar pooled libraries were sequenced on the Illumina NovaSeq 6000 platform (Illumina, San Diego, USA), generating 2× 150 bp paired-end reads.

Short reads generated from the Illumina platform were assessed for quality using FastQC v0.12.1, ensuring that only high-quality reads (Phred score > 30) were retained for downstream analysis. De novo genome assembly was performed using SPAdes^28^. Assembled genomes were uploaded to the Pathogenwatch platform v23.0.2^29^ for comprehensive genotypic analysis. This included Salmonella 7-gene multilocus sequence typing^30^ and genotype identification using Genotyphi^31^. Additionally, antimicrobial resistance (AMR) genes, point mutations, plasmid replicon types, and single nucleotide polymorphisms (SNPs) were detected within the Pathogenwatch framework.

## Data Availability

The deidentified datasets used in this study are available from the corresponding author upon request. The whole-genome sequence reads of the S. Paratyphi B isolates are available from the Sequence Read Archive (SRA) database using the following accession numbers: SRR31644347, SRR31644344, and SRR31644343. The whole-genome sequence reads of the ceftriaxone resistant S. Typhi isolates are available from the European Nucleotide Archive (ENA) database using the following run accession numbers: ERR12652117, ERR12652116, ERR12652115, ERR12652114, ERR12652113, ERR12652112, ERR12652111, ERR12652110, ERR12652108, ERR12081858, ERR11458680, ERR11458681, ERR11458682, ERR11458674, and ERR11458676.

## References

[1] Institute for Health Metrics and Evaluation. *Typhoid and Paratyphoid—Level 3 Cause*. https://www.healthdata.org/research-analysis/diseases-injuries-risks/factsheets/2021-typhoid-and-paratyphoid-level-3-disease.

[2] Garrett, D. O. et al. Incidence of typhoid and paratyphoid fever in Bangladesh, Nepal, and Pakistan: Results of the surveillance for enteric fever in Asia project. *Lancet Glob. Health* **10**, e978–e988 (2022). 35714648 10.1016/S2214-109X(22)00119-X PMC9210262

[3] Cao, Y. et al. Geographic pattern of typhoid fever in India: A model-based estimate of cohort and surveillance data. *J. Infect. Dis.* **224**(Suppl 5), S475–S483 (2021). 35238365 10.1093/infdis/jiab187 PMC8892532

[4] Butt, M. H. et al. Rising XDR-typhoid fever cases in Pakistan: Are we heading back to the pre-antibiotic era?. *Front. Public Health* **9**, 794868 (2021). 35111719 10.3389/fpubh.2021.794868 PMC8801676

[5] Ahmad, S. et al. A skeleton in the closet: The implications of COVID-19 on XDR strain of typhoid in Pakistan. *Public Health Pract. (Oxf.)* **2**, 100084 (2021). 33521736 10.1016/j.puhip.2021.100084 PMC7826057

[6] Taneja, N. & Sharma, M. Antimicrobial resistance in the environment: The Indian scenario. *Indian J. Med. Res.* **149**, 119–128 (2019). 31219076 10.4103/ijmr.IJMR_331_18 PMC6563737

[7] Sulis, G., Batomen, B., Kotwani, A., Pai, M. & Gandra, S. Sales of antibiotics and hydroxychloroquine in India during the COVID-19 epidemic: An interrupted time series analysis. *PLoS Med.* **18**, e1003682 (2021). 34197449 10.1371/journal.pmed.1003682 PMC8248656

[8] Ali, S. et al. Emergence of extensive drug resistance typhoid in hospitalized COVID-19 patients in South Punjab, Pakistan. *J. Popul. Ther. Clin. Pharmacol.* **31**, 753–763 (2024).

[9] Malorny, B., Bunge, C. & Helmuth, R. Discrimination of d-tartrate-fermenting and -Nonfermenting *Salmonella enterica* subsp. *enterica* isolates by genotypic and phenotypic methods. *J. Clin. Microbiol.* **41**, 4292–4297 (2003). 12958259 10.1128/JCM.41.9.4292-4297.2003 PMC193836

[10] Clinical and Laboratory Standards Institute. *Performance Standards for Antimicrobial Susceptibility Testing* 33rd edn. (CLSI supplement M100, Standards Inst, 2023).

[11] Veeraraghavan, B. et al. Evaluation of antimicrobial susceptibility profile in *Salmonella* Typhi and *Salmonella* Paratyphi A: Presenting the current scenario in India and strategy for future management. *J. Infect. Dis.* **224**(Suppl 5), S502–S516 (2021). 35238369 10.1093/infdis/jiab144 PMC8892543

[12] Dahiya, S. et al. Current antibiotic use in the treatment of enteric fever in children. *Indian J. Med. Res.* **149**, 263–269 (2019). 31219092 10.4103/ijmr.IJMR_199_18 PMC6563751

[13] Fazaludeen Koya, S., Hasan Farooqui, H., Mehta, A., Selvaraj, S. & Galea, S. Quantifying antibiotic use in typhoid fever in India: a cross-sectional analysis of private sector medical audit data, 2013–2015. *BMJ Open* **12**, e062401 (2022). 36253043 10.1136/bmjopen-2022-062401 PMC9577907

[14] Sah, R. et al. A novel lineage of ceftriaxone-resistant *Salmonella* Typhi from India that is closely related to XDR *S.* Typhi Found in Pakistan. *Clin. Infect. Dis.* **71**, 1327–1330 (2020). 31872221 10.1093/cid/ciz1204

[15] Dzeyie, K. A. et al. Outbreak of ceftriaxone-resistant *Salmonella enterica* serotype Typhi-Tiruchirappalli, Tamil Nadu, India, June 2018. *IJID Reg.* **1**, 60–64 (2021). 35757827 10.1016/j.ijregi.2021.09.006 PMC9216270

[16] Samajpati, S., Pragasam, A. K., Mandal, S., Balaji, V. & Dutta, S. Emergence of ceftriaxone resistant *Salmonella enterica* serovar Typhi in Eastern India. *Infect. Genet. Evol.* **96**, 105093 (2021). 34592414 10.1016/j.meegid.2021.105093

[17] Argimón, S. et al. Circulation of third-generation cephalosporin resistant *Salmonella* Typhi in Mumbai, India. *Clin. Infect. Dis.* **74**, 2234–2237 (2022). 34626469 10.1093/cid/ciab897 PMC9258936

[18] Jacob, J. J. et al. *Salmonella* Typhi acquires diverse plasmids from other Enterobacteriaceae to develop cephalosporin resistance. *Genomics* **113**(4), 2171 (2021). 33965548 10.1016/j.ygeno.2021.05.003 PMC8276774

[19] Sharma, P. et al. Azithromycin resistance mechanisms in typhoidal salmonellae in India: A 25 years analysis. *Indian J. Med. Res.* **149**, 404–411 (2019). 31249207 10.4103/ijmr.IJMR_1302_17 PMC6607824

[20] Central Bureau of Health Intelligence-Government of India. *National Health Profile 2022* (Central Bureau of Health Intelligence-Government of India, 2022). https://cbhidghs.mohfw.gov.in/WriteReadData/l892s/94203846761680514146.pdf.

[21] Central Bureau of Health Intelligence-Government of India. *National Health Profile 2021* (Central Bureau of Health Intelligence-Government of India, 2021). https://ruralindiaonline.org/en/library/resource/national-health-profile-2021/.

[22] Sonal, S., Anuj, S., Amala, A. A., WINSAR-D Network members. Delhi’s network for surveillance of antimicrobial resistance: The journey, challenges and output from first year. *Indian J. Med. Microbiol.* **41**, 19–24 (2023). 36870743 10.1016/j.ijmmb.2022.12.001

[23] Chauhan, A. S. et al. Cost effectiveness of typhoid vaccination in India. *Vaccine* **39**(30), 4089–4098 (2021). 34120765 10.1016/j.vaccine.2021.06.003 PMC8256879

[24] Nampota-Nkomba, N., Carey, M. E., Jamka, L. P., Fecteau, N. & Neuzil, K. M. Using typhoid conjugate vaccines to prevent disease, promote health equity, and counter drug-resistant typhoid fever. *Open Forum Infect. Dis.* **10**(Suppl 1), S6–S12 (2023). 37274532 10.1093/ofid/ofad022 PMC10236511

[25] John, J. et al. Burden of typhoid and paratyphoid fever in India. *N. Engl. J. Med.* **388**, 1491–1500 (2023). 37075141 10.1056/NEJMoa2209449 PMC10116367

[26] Carey, M. E. et al. The surveillance for enteric fever in Asia project (SEAP), severe typhoid fever surveillance in Africa (SETA), surveillance of enteric fever in India (SEFI), and strategic typhoid alliance across Africa and Asia (STRATAA) population-based enteric fever studies: A review of methodological similarities and differences. *Clin. Infect. Dis.* **71**(Suppl 2), S102–S110 (2020). 32725221 10.1093/cid/ciaa367 PMC7388711

[27] Versalovic, J. et al. (eds) *Manual of Clinical Microbiology* 10th edn. (American Society for Microbiology, 2011).

[28] Prjibelski, A., Antipov, D., Meleshko, D., Lapidus, A. & Korobeynikov, A. Using SPAdes De Novo Assembler. *Curr. Protoc. Bioinforma.* **70**(1), e102 (2020). 10.1002/cpbi.102 32559359

[29] Centre for Genomic Pathogen Surveillance. *Pathogenwatch*. https://pathogen.watch/. Accessed 18 Jan 2025.

[30] Achtman, M. et al. Multilocus sequence typing as a replacement for serotyping in *Salmonella enterica*. *PLoS Pathog.* **8**(6), e1002776 (2012). 22737074 10.1371/journal.ppat.1002776 PMC3380943

[31] Dyson, Z. A. & Holt, K. E. Five years of GenoTyphi: Updates to the global *Salmonella* Typhi genotyping framework. *J. Infect. Dis.* **224**(Suppl 7), S775–S780 (2021). 34453548 10.1093/infdis/jiab414 PMC8687072

